# Role of Novel Target Molecules (β-Catenin and Retinoblastoma Protein) in the Spectrum of Inflammatory Bowel Disease

**DOI:** 10.7759/cureus.83162

**Published:** 2025-04-29

**Authors:** Manali V Bharambe, Preeti R Doshi, Rachana R Lakhe, Purva S Kulkarni, Reena P Bharadwaj

**Affiliations:** 1 Pathology, Bharati Vidyapeeth (Deemed to be University) Medical College, Pune, Pune, IND

**Keywords:** crohn’s disease, inflammatory bowel disease, retinoblastoma protein, ulcerative colitis, β-catenin

## Abstract

Background

Inflammatory bowel disease (IBD) has been a worldwide health care challenge with a continually increasing incidence in India. Spectrum of IBD mainly includes ulcerative colitis (UC) and Crohn’s disease (CD). Pharmacological and surgical treatments differ widely in CD and UC; hence, establishment of a correct diagnosis is of paramount importance as it critically influences the disease outcome.

Aim

The aim of this study was to assess the utility and diagnostic accuracy of immunohistochemical profile of β-catenin and retinoblastoma protein in IBD as potential biomarkers.

Study design

This is a cross-sectional observational study.

Material and methods

Data of 61 cases of IBD with UC and CD were retrieved from the Department of Pathology. Immunohistochemical markers β-catenin and retinoblastoma protein were applied on the paraffin blocks of these cases, and their results were interpreted.

Results

Of the 61 cases, 49 cases of UC showed positivity for β-catenin with 100% sensitivity and 75% specificity and nine cases of CD showed positivity for retinoblastoma protein with 75% sensitivity and 95.92% specificity. Three cases were IBD unclassified. Overall, accuracy of β-catenin in UC was found to be 95.08% and that of retinoblastoma protein in CD was 91.80%.

Conclusion

Our study provides insights into the expressions of these specific immunohistochemistry markers and their significance in the differentiation of IBD into UC and CD.

## Introduction

Inflammatory bowel disease (IBD) is continuously increasing worldwide. The two main disorders under IBD are ulcerative colitis (UC) and Crohn’s disease (CD). A multifactorial pathogenesis is seen in IBD, including epithelial barrier defects, genetic predisposition, dysregulated immune responses, and environmental factors, contributing to the onset of the disease and its progression [1].

UC and CD differ from each other in disease course and have different complications and management. The recognition of correct histopathological findings should be done to differentiate UC and CD [2]. The differentiation between these two entities can be challenging due to overlap of microscopic features on histopathology. As the pharmacological and surgical management differs in UC and CD, the establishment of a correct diagnosis is very important [3].

Till today, many studies are available on IBD but only few have focused on the two important regulators - β-catenin and retinoblastoma protein - playing crucial role in proliferation of colonic tissue and inflammation [4].

β-catenin plays an important role in IBD as it is a protein encoding the CTNNB1 gene with functions such as Wnt signaling and cell adhesion. Lymphoid enhancer-binding factor/T-cell factor has also found to mediate the function of β-catenin in the nucleus. It plays a role in both signaling and adhesiveness due to its structural composition, making it bind at nuclear, membranous, and cytoplasmic levels [5]. In normal tissue, beta-catenin has cytoplasmic or membranous staining. In epithelial cells, it shows positivity at the baso-lateral part of the membrane. However, progenitor cells at the bottom of colonic crypts show nuclear or cytoplasmic β-catenin positivity to drive proliferation by binding to T-cell factor/lymphoid enhancer factor [3].

Retinoblastoma protein is named after retinoblastoma, a retinal tumor seen in childhood. It is a tumor suppressor gene at 13q14. Being a nuclear protein, it plays important role in the regulation of arrest in cell cycle at the level of G1-S phase [6]. The retinoblastoma protein shows positivity in colonic inflammation. Due to inflammation, there is release of TNF-a, leading to cell death, and RB protein has shown to have protection against the death of intestinal epithelial cells [3].

## Materials and methods

A total of 61 patients diagnosed with IBD were included in this cross-sectional observational study. Of the 61 patients, 49 were diagnosed with UC and 12 with CD. Males (32) were found to be more affected than females (29) in this study, with a mean age of 42 years (range: 11-72 years). The duration of the study was one year, starting from June 2023 to May 2024. All colonic biopsies diagnosed as IBD by histopathological examination were included, and colon biopsies with non-specific colitis and colonic adenocarcinomas were excluded. The study was approved by the Institutional Ethics Committee of Bharati Vidyapeeth (Deemed to be University) Medical College, Pune (approval number BVDUMC/IEC/35).

**Figure 1:**
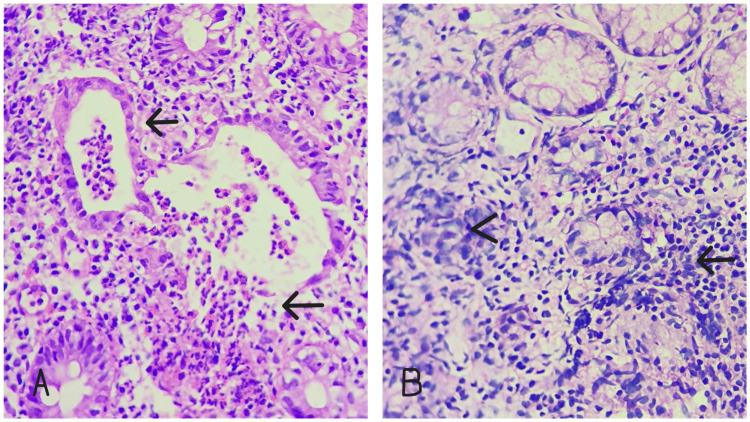
H&E stain. (A) Ulcerative colitis showing crypt dilatation and crypt abscess (black arrows). (B) Crohn’s disease showing granuloma (arrowhead) and basal plasmacytosis (black arrow).

Patients presented with chronic diarrhea, per rectal bleeding, pain in the abdomen, and weight loss. Biopsies of the affected part of the colon and rectum were included in this study, and diagnosis of IBD was made by histopathological examination (Figure 1).

Serial sections and slides were made to apply immunohistochemistry (IHC) markers. Master Polymer Plus Detection System kit (Vitro Master Diagnostica, Granada, Spain) was used as per the manufacturer’s instructions. Normal colon and breast carcinoma tissue were taken as positive IHC controls for β-catenin and retinoblastoma protein, respectively.

**Figure 2:**
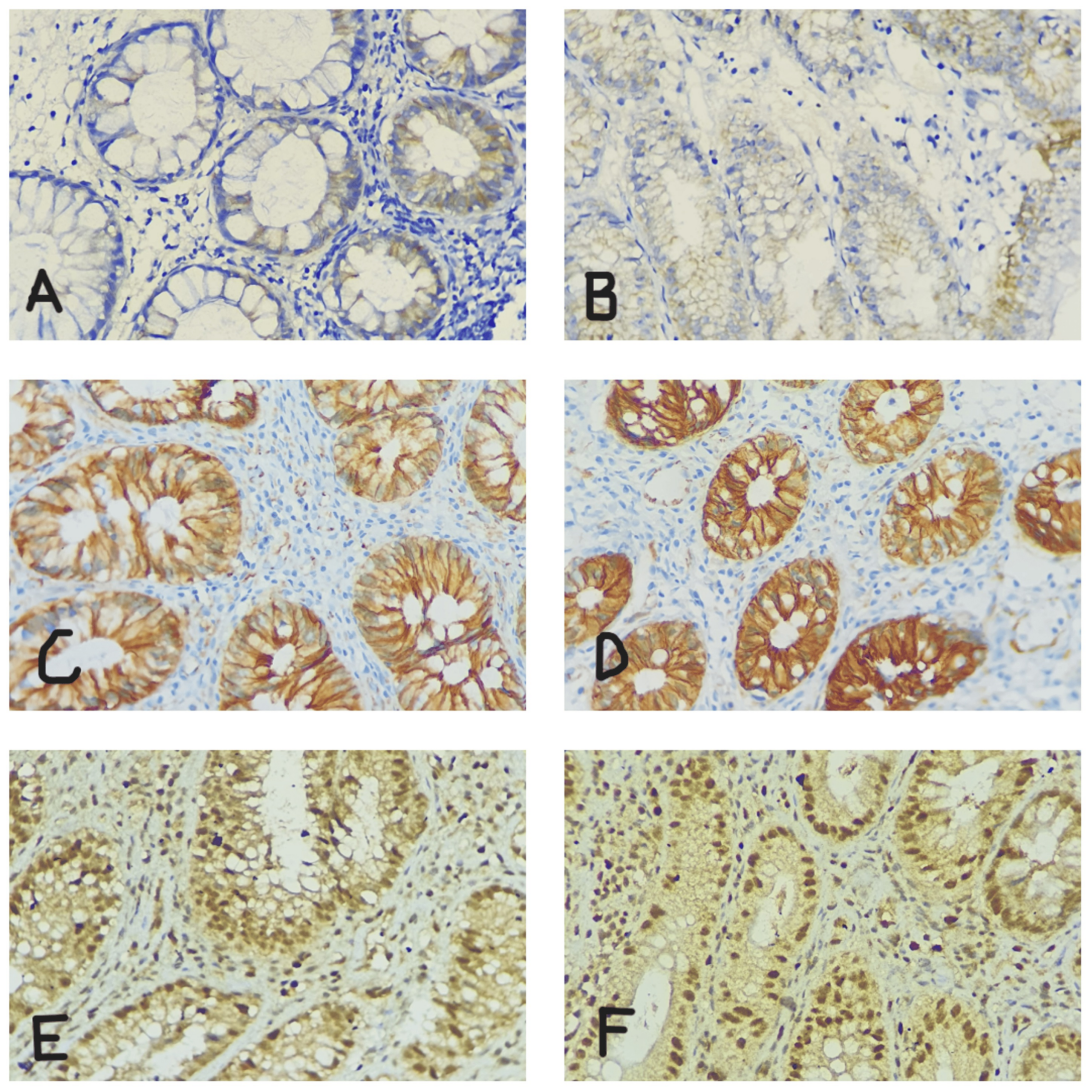
Immunohistochemistry. Intensity scoring of β-catenin: (A) minimal membrane positivity (score 0), (B) mild membrane positivity (score 1), (C) moderate membrane positivity (score 2), and (D) strong cytoplasmic and membranous positivity (score 3). Percentage of retinoblastoma protein positivity in (E) 50-60% and (F) >90%.

Epithelial cells showing reactivity and positivity distinct from the background were noted (Figure 2). Around 10-20 crypts from each colon biopsy sample were examined, and the scoring was done. β-catenin was examined and categorized according to intensity scoring at the nucleus and according to cytoplasmic and cell membrane positivity (0, negative; 1, mild; 2, moderate; 3, strong). Retinoblastoma scoring was done in percentage.

Statistical software SPSS (Statistical Package for Social Science) Version 26.0 (IBM Corp., Armonk, NY) was used. Discrimination between UC and CD was carried out in terms of sensitivity, specificity, positive predictive values, negative predictive values, and accuracy based on histopathological examination as the gold standard for diagnosis. Approval from the Institutional Ethical Committee was taken.

## Results

Out of 61 cases, 49 cases of UC showed positivity for β-catenin. β-catenin was found to have potential involvement in UC than CD after studying the colonic biopsies with 100% sensitivity and 75% specificity (Table 1). β-catenin accumulation was found in the cell membrane, cytoplasm, or nucleus of cases with UC and was scored according to the staining intensity.

**Table 1:** Statistical analysis for β-catenin Asterisk (*) depicts the statistical significance at 0.05 level (p<0.05)

Statistic	Value	95% CI
Sensitivity	100.00%	92.75% to 100.00%
Specificity	75.00%	42.81% to 94.51%
Positive predictive value (*)	94.23%	85.97% to 97.75%
Negative predictive value (*)	100.00%	66.37% to 100.00%
Accuracy (*)	95.08%	86.29% to 98.97%

Retinoblastoma protein phosphorylation was seen in high percentages in patients with CD than UC. Nine cases of CD showed positivity for retinoblastoma protein with 75% sensitivity and 95.92% specificity (Table 2). Overall, accuracy of β-catenin in UC was found to be 95.08% and that of retinoblastoma protein in CD was 91.80%.

**Table 2:** Statistical analysis for retinoblastoma protein Asterisk (*) depicts the statistical significance at 0.05 level (p<0.05)

Statistic	Value	95% CI
Sensitivity	75.00%	42.81% to 94.51%
Specificity	95.92%	86.02% to 99.50%
Positive predictive value (*)	81.82%	52.70% to 94.79%
Negative predictive value (*)	94.00%	85.44% to 97.66%
Accuracy (*)	91.80%	81.90% to 97.28%

Three cases had IBD unclassified as they all showed high expressions of both retinoblastoma protein and β-catenin. The patients had chief complaints of chronic diarrhea, abdominal pain, and per rectal bleeding. On histopathological examination, no specific findings of UC or CD were noted, and on application of IHC, both the markers (beta catenin and retinoblastoma protein) were positive. Therefore, three such cases were put under the category of IBD unclassified.

This study shows significant differences in the expression levels of β-catenin and retinoblastoma protein in patients with IBD and also helps assess the utility and diagnostic accuracy of immunohistochemical profile of β-catenin and retinoblastoma protein in IBD as potential biomarkers.

## Discussion

CD and UC show substantial differences in disease course, suggesting distinct etiopathogenic processes [7,8]. A multifactorial pathogenesis is seen in IBD, including epithelial barrier defects, genetic predisposition, dysregulated immune responses, and environmental factors, contributing to the onset of the disease and its progression [1]. UC and CD differ from each other in disease course and have different complications and management [3].

Earlier, IHC marker studies and molecular studies have been conducted to know the events involved in pathogenesis of the disease, and many investigations for susceptibility genes and protein expression analysis have been carried out [9]. Several oxidative stress inducers and inflammatory mediators have also been found to play a role in this disease [1].

The study conducted by Ying et al. has shown the interference of retinoblastoma protein function by the proinflammatory markers and macrophages inhibitory factors in patients with colitis, causing hyperphosphorylation of retinoblastoma protein and release of E2F1 activating genes, further causing proliferation and inhibition of apoptosis. This showed an association between chronic inflammation and cancer due to involvement in the activation of the same pathways [6].

In the study by Lawrance et al., inflamed colonic tissues were assessed by DNA microarrays for global gene expression profiles. These showed significant differences in the expression profiles, identifying IBD into UC and CD as distinct molecular entities by studying expression profiles of 170 genes [10].

The study conducted by Derkacz et al. showed the use of biomarkers as helpful tool for diagnosis. It was also useful for prognosis from a clinical point of view. They used fecal calprotectin and lactoferrin in their study and stated that increase in the diagnostic specificity and sensitivity of IBD is due to development of accurate non-invasive biomarkers. They also stated that extracellular matrix components, when used as biomarkers, can help evaluate early gut changes in IBD patients [11].

Study on immunological markers was conducted in view of microbial aberrant immune response or endogenous antigens in a host that is genetically susceptible involved in pathogenesis of IBD [12-14]. Different microbial antigens and their antibodies, along with IBD associated autoantibodies targeting the exocrine pancreas (PAB), have shown high specificity for CD. This study showed that these immunological markers are important as a differential diagnosis of UC versus CD [15,16].

In this study, we found significance differences in the expression levels of β-catenin and retinoblastoma protein in cases of IBD. These two IHC markers were applied on colonic biopsies and studied for the accumulation of proteins. β-catenin was found to be increased in UC, and retinoblastoma protein was found to be increased in CD.

Retinoblastoma protein being a tumor suppressor has a role in controlling cell cycle. In CD, retinoblastoma protein phosphorylation is seen increased following inflammatory stimuli [6]. β-catenin imbalance often results in disease and deregulated growth of cancer cells due to its signaling properties and structure. In UC, β-catenin nuclear/cytoplasmic translocation is seen to happen in the presence of inflammation [5].

To know the molecular events involving IBD pathogenesis with difference in UC and CD, many research groups have analyzed expressions of protein and susceptibility of various genes [17]. Abraham and Cho studied expressions of genes such as NOD2 and the autophagy gene, ATG16L1, which played major roles in the pathogenesis of CD and not UC, differentiating it from the later. Also, in UC, significance of genome-associated studies was observed, with MHC class II region being present near HLA DRA (alpha chain) [18,19].

Till today, not a single study has been conducted with just two IHC markers addressing the differences in the expressions of β-catenin and retinoblastoma protein to differentiate UC from CD. Accurate differentiation on histopathology is challenging due to overlap in histologic features, and thus this study will be useful for disease treatment and prognosis as both the markers play a crucial role in inflammation and epithelial cell proliferation [20].

Limitations

Three cases showed high expressions of both retinoblastoma protein and β-catenin. Therefore, further studies with larger cohorts are needed to use these immunohistochemical markers on routine basis for the differentiation of IBD into UC and CD. Also, the region where this study was conducted had less cases of CD compared to UC for fair comparison between two entities.

## Conclusions

Histopathology is considered the primary examination to identify IBD, but due to overlapping histological features, it is often difficult to distinguish UC from CD. β-catenin and retinoblastoma protein are found to play important roles as regulators of IBD. Among all the new studies with advance molecular mechanisms, not a single study depicts the use of immunohistochemistry on a routine basis for distinguishing IBD into UC and CD. Our study provides insights into the expressions of these specific IHC markers and their significance in the differentiation of IBD into UC and CD. This is important as different lines of treatments are present for both the entities resulting in better patient care and the disease outcome.
